# Heterologous Metabolic Pathways: Strategies for Optimal Expression in Eukaryotic Hosts

**DOI:** 10.32607/actanaturae.10966

**Published:** 2020

**Authors:** N. M. Markina, A. A. Kotlobay, A. S. Tsarkova

**Affiliations:** Shemyakin-Ovchinnikov Institute of Bioorganic Chemistry of the Russian Academy of Sciences, Moscow, 117997 Russia; Planta LLC, Moscow, 121205 Russia; Pirogov Russian National Research Medical University, Moscow, 117997 Russia

**Keywords:** metabolic pathways, heterologous expression, secondary metabolites

## Abstract

Heterologous pathways are linked series of biochemical reactions occurring in a host organism after the introduction of foreign genes. Incorporation of metabolic pathways into host organisms is a major strategy used to increase the production of valuable secondary metabolites. Unfortunately, simple introduction of the pathway genes into the heterologous host in most cases does not result in successful heterologous expression. Extensive modification of heterologous genes and the corresponding enzymes on many different levels is required to achieve high target metabolite production rates. This review summarizes the essential techniques used to create heterologous biochemical pathways, with a focus on the key challenges arising in the process and the major strategies for overcoming them.

## INTRODUCTION

Today, incorporation of metabolic pathways into host organisms is a major strategy for increasing the production of valuable secondary metabolites. Heterologous expression began as the introduction of a single foreign gene into the cells of host organisms, termed expression systems, most of which at the time were bacteria. Over the past 40 years, the methodology of heterologous gene expression has significantly evolved, making it possible to introduce both individual genes and entire gene clusters into the genomes of various host organisms [1,2]. The development of new methods of heterologous expression of gene clusters has spawned a new field - metabolic engineering, successful application of which requires large-scale analysis and manipulation of various biochemical pathways that form interconnected networks [3].

This paper reviews the essential techniques for creating heterologous biochemical pathways in various host organisms, outlines some key challenges arising in the process, and suggests some strategies for overcoming them.

## MODERN TECHNIQUES OF METABOLIC ENGINEERING

**Fig. 1:**
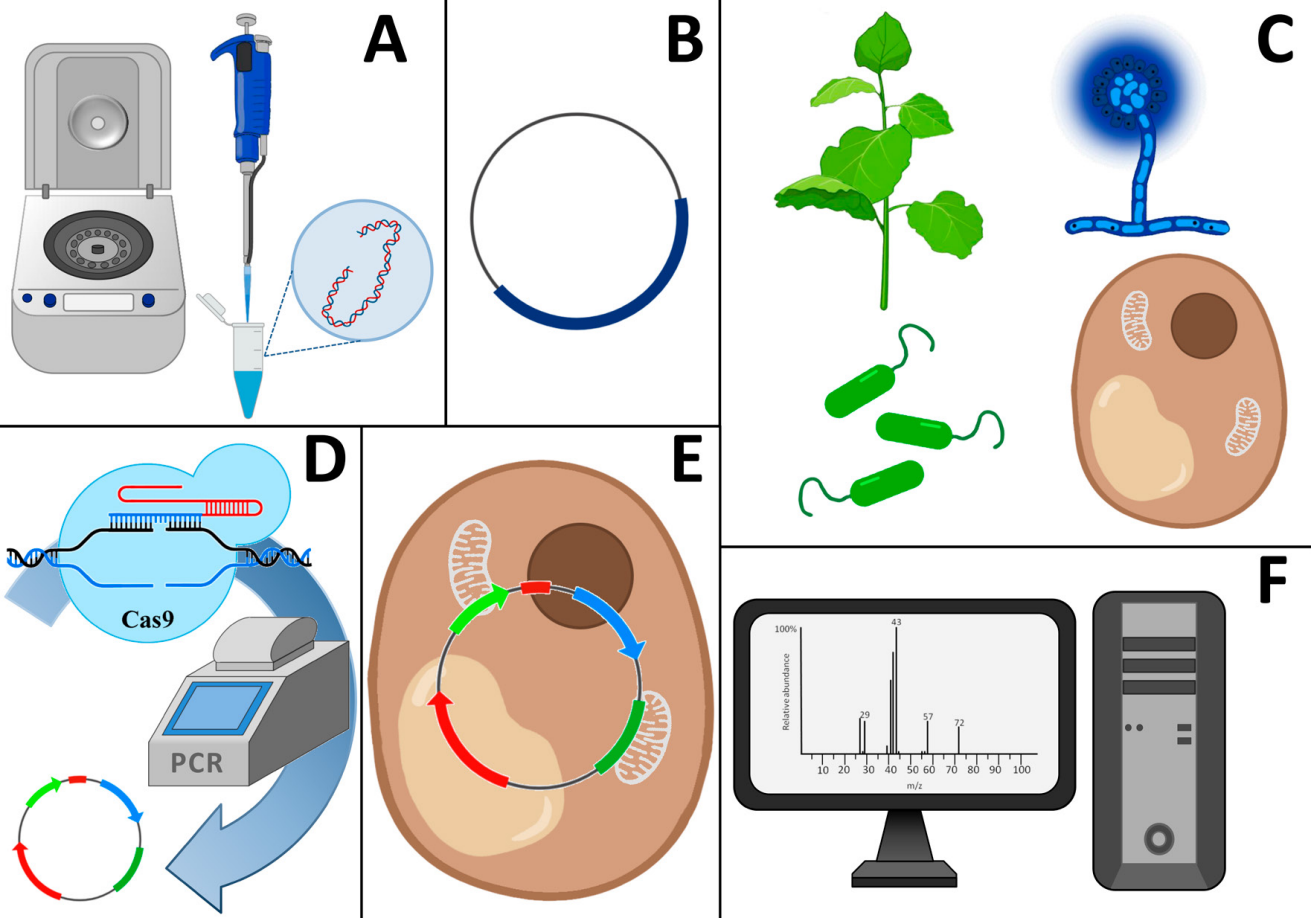
The typical workflow for heterologous expression of metabolic pathways.* A *– DNA isolation from a native producer;* B *– insertion of DNA into vectors;* C *– appropriate heterologous host selection;* D *– genetic manipulations;* E *– vector maintenance in heterologous host;* F *– optimization of metabolite production

Although modern metabolic engineering techniques have permitted us to acquire multiple biologically derived chemicals, there is no single approach yet that would result in successful heterologous expression. The following key steps must be taken to efficiently insert an exogenous metabolic pathway into a heterologous host:

1. Isolation of the necessary metabolic pathway genes for the biosynthesis of the target compound;

2. Incorporation of the biosynthetic pathway genes into a suitable stable vector(s);

3. Selection of an appropriate host organism; and

4. Selection of methods for the maintenance and optimization of the given metabolic pathway in the heterologous host [4] (*Fig. 1*).

Even if all these conditions are met, it is almost impossible to predict in advance whether functional heterologous expression of a gene cluster will be achieved. In some cases, the heterologous metabolic pathway works with virtually no additional modifications, while a lengthy and extensive optimization is required for other pathways and organisms [5-8].

Alongside the experimental approaches, computational and modeling methods for the elucidation of metabolic pathways and their manipulation in host cells have been developed. The *in silico *models are highly predictive when applied to well-investigated metabolic pathways and well-known host organisms. Computational models allow researchers to alter gene expression and enzyme production levels *in silico *and directly observe their effect on the pathway flux. These models, however, are difficult, if not impossible, to apply to experimental systems for which many crucial parameters are unknown [9]. A broader bioinformatics approach is the creation of metabolic models of whole organisms [10,11]. In addition to their fundamental value, these models enable the prediction of the availability and quantity of certain metabolites, which, in turn, facilitates the optimal matching of the host and the heterologous metabolic pathway.

In order to incorporate an exogenous metabolic gene cassette into a host organism, one must also take into account the complexity of metabolic networks and the necessity to maintain the metabolic balances in the host; i.e., to monitor the production and consumption of essential metabolites, such as NADH, ATP, and O_2_ [12]. Various computational approaches have been implemented for pathway prediction, with attention focused mainly on the retrosynthetic algorithms generating all possible pathways that link a specific host metabolite to a desired target product [13, 14, 15]. Most retrosynthetic algorithms calculate the shortest heterologous pathways for target metabolites [14,16,17]. This approach, however, is not always optimal, since biochemical reactions commonly require cofactors and pool metabolites, which may be absent or limited in the host organism. In that case, it is preferable to use complex algorithms that factor in the number of participants in each specific reaction [18].

## SELECTION OF A SUITABLE HOST FOR HETEROLOGOUS EXPRESSION

Choosing a suitable expression system for a metabolic pathway is one of the most critical steps in the development of a high-expression process [19]. The most commonly used bacterial expression systems are maintenance-friendly, require low-cost growth media, produce high levels of recombinant proteins, and are accompanied by an array of tools available for their genetic and molecular manipulations [20]. However, in most cases the large size of a heterologous biosynthetic gene cassette and the requirement for the transcriptional and/or posttranslational processing and modifications of foreign proteins make bacterial hosts unsuitable for heterologous expression of complex metabolic pathways [19] or require additional modifications of the host [21]. Fortunately, yeast and fungal protein expression systems are relatively cheap, fast-growing low-maintenance organisms that have proven to be reliable producers of eukaryotic proteins and metabolites. In contrast, insect and mammalian cell lines exhibit slow growth, require special culture conditions, have lower expression levels, and require cumbersome adaptation for metabolic pathway expression. Some advantages and drawbacks for the commonly used eukaryotic heterologous protein expression systems are listed in *Table 1*.

Single-cell eukaryotic microorganisms, yeast, are widely used as hosts for heterologous expression [22]. In addition to the low maintenance requirements, yeast are highly amenable to experimental manipulation via the use of a wide range of readily available metabolic or genetic engineering tools. It is also important that yeast cells contain the necessary molecular machinery for protein folding, are able to carry out the most complex post-translational modifications essential for the proper functioning of eukaryotic enzymes, and can support the functional expression of membrane-anchored enzymes, such as cytochrome P450s. Moreover, the yeasts *P. pastoris* and *S. cerevisiae *have also been given the status of “generally recognized as safe” (GRAS) organisms as they do not produce any known oncogenic or toxic products [23-25].

In particular, *Saccharomyces cerevisiae *is a convenient heterologous host, since an extensive methodology has been developed for controlling the expression of the heterologous biosynthetic pathways in this organism. To become familiar with the general methods of heterologous expression of metabolic pathways in yeast, as well as successful examples of heterologous biosynthesis of secondary metabolites in *S. cerevisiae*, see review [6].

A vast library of constitutive and inducible promoters with varied expression strengths has been described for *Pichia pastoris *(e.g., the methanol-induced promoter of the *alcohol oxidase *I gene (P_AOX1_), which is activated by the addition of methanol and inactivated by the addition of glucose, glycerol or ethanol [26]). If the use of several promoters is required, the availability of various inducible promoters allows one to avoid spontaneous *in vivo *recombination. The existence of sequenced and annotated genomes of several *P. pastoris *strains is also beneficial to metabolic engineering [27]. Moreover, several specialized cloning kits have been developed to facilitate the creation of vectors compatible with *P. pastoris *[28, 29].

Other types of yeast can also be used as heterologous hosts for metabolic pathways, such as the methylotrophic yeasts *Candida boidinii*, *Hansenula polymorpha,* and *Pichia methanolica *[30] and oleaginous yeast *Yarrowia lipolytica *that is able to metabolize crude oil [31, 32].

Among various filamentous fungi, Aspergilli are the most commonly used heterologous hosts [19]. The undisputed advantages of fungi include the simplicity of cultivation and the rapid growth of biomass [33]. The use of *Aspergillus *species as hosts can be extremely convenient for the heterologous expression of fungal gene clusters, since source promoters and terminators can be exploited. For example, a cluster of penicillin biosynthesis genes was successfully transferred to* Neurospora crassa *and *Aspergillus niger *[34]. However, in some cases, in order to increase the metabolite production, the original regulatory sequence still needs to be replaced with the promoter of the host organism, since the exogenous ones tend to be relatively weak and/or can only be expressed under certain specific conditions [22, 35]. For general information on strategies of heterologous expression of metabolic pathways in Aspergilli see [36].

Plants are a promising expression system for the heterologous production of plant natural products [37]. Metabolic engineering of plants is especially justified when the target metabolic pathway includes large and poorly transferable enzymes, since many plant biosynthetic pathways require post-translational modification, coenzymes, co-factors, or regulators and are compartmentalized in specific subcellular organelles [38].

When working with plants, it is important to understand that their metabolism varies significantly depending on the species, the tissue and the developmental stage; often the same plant changes its metabolic profile almost beyond recognition during flowering [39]. The strategies used for plant metabolic engineering were reviewed in [40].

**Table 1:** Summary of the suitable eukaryotic hosts for heterologous expression

Host	Benefits	Handicaps	Common species
Yeast	Low-maintenance fast-growing single-cell organisms High protein expression levels Easy regulation of cell mating type (sexual or asexual) Possess typical enzymes for protein-folding and post-translational modifications Availability of robust genetic manipulation tools Ability to express membrane enzymes and secretion proteins Generally recognized as safe (GRAS) (i.e., do not produce highly toxic or oncogenic substances)	Potential hyperglycosylation at N-linked sites, which may reduce protein function Tough cell wall Low diversity of native secondary metabolites, hindering the selection of suitable precursors	Saccharomyces cerevisiae Pichia pastoris (Komagataella) Candida boidinii, Hansenula polymorpha, Pichia methanolica Yarrowia lipolytica
Filamentous fungi	Low-maintenance fast-growing cultures High diversity of native secondary metabolites, facilitating the selection of suitable precursors	Abundance of native metabolic pathways: production of the desired metabolite is forced to compete with the metabolism of the host Spores hazardous to health Limited expression levels	Aspergillus spp., Neurospora crassa
Plants	Well suited for heterologous expression of metabolic pathways from other plants Expression of large enzymes Host versatility: whole organism or a cell culture The heterologous metabolic pathway can be localized in the chloroplasts	High cost of engineering and cultivation Complex transformation protocols Low growth and reproduction rates	Nicotiana benthamiana, Nicotiana tabacum, Arabidopsis thaliana, Physcomitrella patens, Chlamydomonas reinhardtii
Animal cell cultures	Highly efficient viral transduction methods Efficient for expression of enzymes derived from other animals (including specific protein modifications) Absence of the cell wall, which is good for product purification	High cost of cultivation Require specific cultivation conditions and complicated equipment Low growth rate	Mammalian cells Insect cells

It is noteworthy that plants can be used as an expression system both in the form of a whole organism and as a cell culture, each having its own advantages: the whole organism is self-sufficient and requires minimal maintenance from the researcher, while the cell culture usually yields higher quantities of target metabolites [38]. At present, the more primitive plants (mosses and algae) are particularly attractive as a source of cell cultures [41].

Chloroplasts, the semiautonomous organelles in plant cells, serve as biosynthetic sites for various metabolites. These organelles have a double membrane and are characterized by a high concentration of ATP and a variety of low-molecular-weight compounds, which makes them another promising bioengineering target. Studies have shown that localization of the heterologous pathway in the chloroplasts typically significantly increases production of the target metabolite [42, 43].

The disadvantages of plants as heterologous hosts include the relatively high cost of engineering, complex transformation protocols, slow growth and reproduction rates, as well as the negative public attitude towards genetically modified plants.

## MODERN METHODS OF VECTOR ENGINEERING

**Fig. 2:**
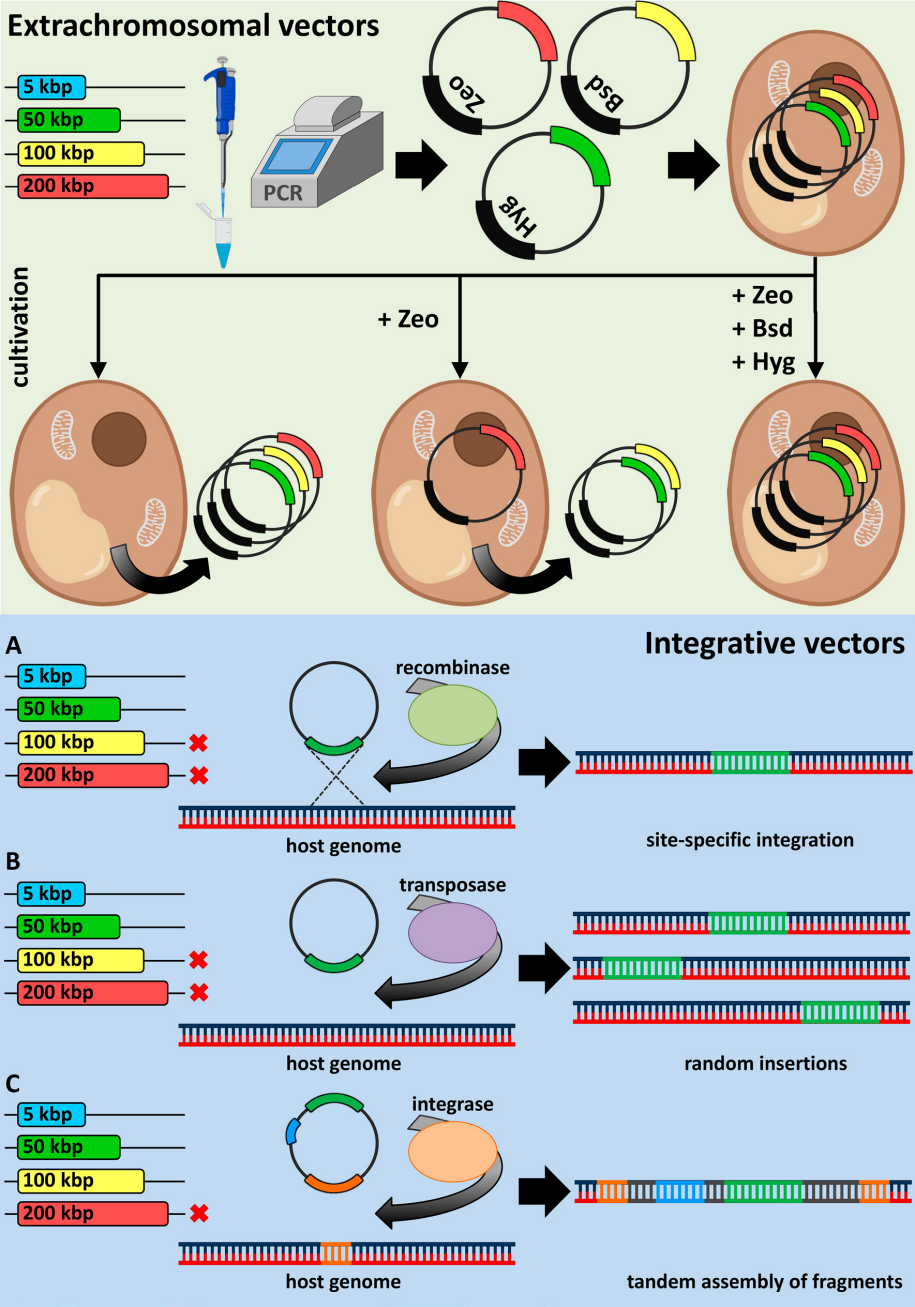
Integrative and non-integrative gene delivery tools. Schematic representation of the incorporation mechanisms of extrachromosomal (top) and integrative (bottom) vectors in host cells

The selection of the necessary vector for gene transfer of the metabolic pathway is largely determined by the host organism in which heterologous expression is planned. A vector must be able to efficiently transduce its target cells, as well as stably replicate in the selected host either by incorporation into the genome or as extragenomic DNA [44]. Furthermore, the genes encoded in it must be efficiently transcribable [4]. Expression vectors can be classified into two general categories: extrachromosomal and integrative (*Fig. 2*).

**Extrachromosomal vectors**

Extrachromosomal genetic elements known as plasmids were first developed as a vector system for bacteria over 40 years ago [45,46]. Today, plasmid vectors are widely recognized as a pivotal tool in the field of metabolic engineering of various microorganisms. The advantages of plasmid constructs are their ease of assembly and manipulation using common methods of molecular biology, and a sufficiently large genetic capacity. The disadvantages of plasmid vectors include the possibility of their spontaneous recombination in the host organism, the need for continuous selective pressure in order to prevent plasmid loss, and the need to employ a large number of different selective markers if several plasmids are used [4].

The recent development of the Modular Cloning System and the availability of commercial standard parts has significantly streamlined the engineering of extrachromosomal plasmids for yeast, thus permitting the assembly of both low- and high-copy plasmids with either single or several coding sequences [47].

**Integrative vec**

Direct incorporation of biosynthetic gene cassettes into the host genome is an alternative approach to heterologous gene delivery. The main methods of chromosomal integration are based on recombination, transposition, or viral-mediated integration of exogenous genomes into the host DNA [4].

Vectors containing exogenous target genes flanked with the host recombination sites are used for homologous recombination. The endogenous host recombinases promote the site-specific integration of target genes into the chromosome of the heterologous host. However, the efficiency of homologous recombination is greatly dependent on the size of the gene cassette. Therefore, successful integration and expression of a large metabolic pathway might require several sequential recombination steps [48].

Gene delivery based on transposition recruits the so-called “jumping genes”, transposons, and the transposase enzyme, which recognizes the specific flanking sites of the target gene cassette. Longer gene sequences are transposed less efficiently; however, unlike in the case of homologous recombination, the insertion sites of transposons are random, resulting in varying levels of heterologous expression from clone to clone and allowing one to select the clones with superior target metabolite production rates [49]. An additional advantage of transposons is the omnitude of the same plasmid construct for multiple hosts [4].

The viral-mediated gene delivery system is based predominantly on bacteriophage integrases and the corresponding integration sequences: thus, many methods are based on φC31 integrase [50, 51], derived from φBT1 [52, 53], or comprise several integrases [54]. Integrase systems can provide means for the efficient integration of large DNA sequences (up to 100 kbp) into specific genomic loci, allowing for iterative or one-step tandem assembly of fragments [52]. The obvious drawback of the system is that a specific molecular machinery (i.e., integrases and auxiliary enzymes) is required.

Irrespective of the chosen DNA delivery method, attention should be paid to the coding sequences being incorporated. They may be either directly obtained from the source organisms or chemically synthesized. The latter option is preferred, because it also makes it possible to optimize the codon content [2, 4], thus improving the heterologous gene expression level [25].

## HETEROLOGOUS PATHWAY OPTIMIZATION

**Fig. 3:**
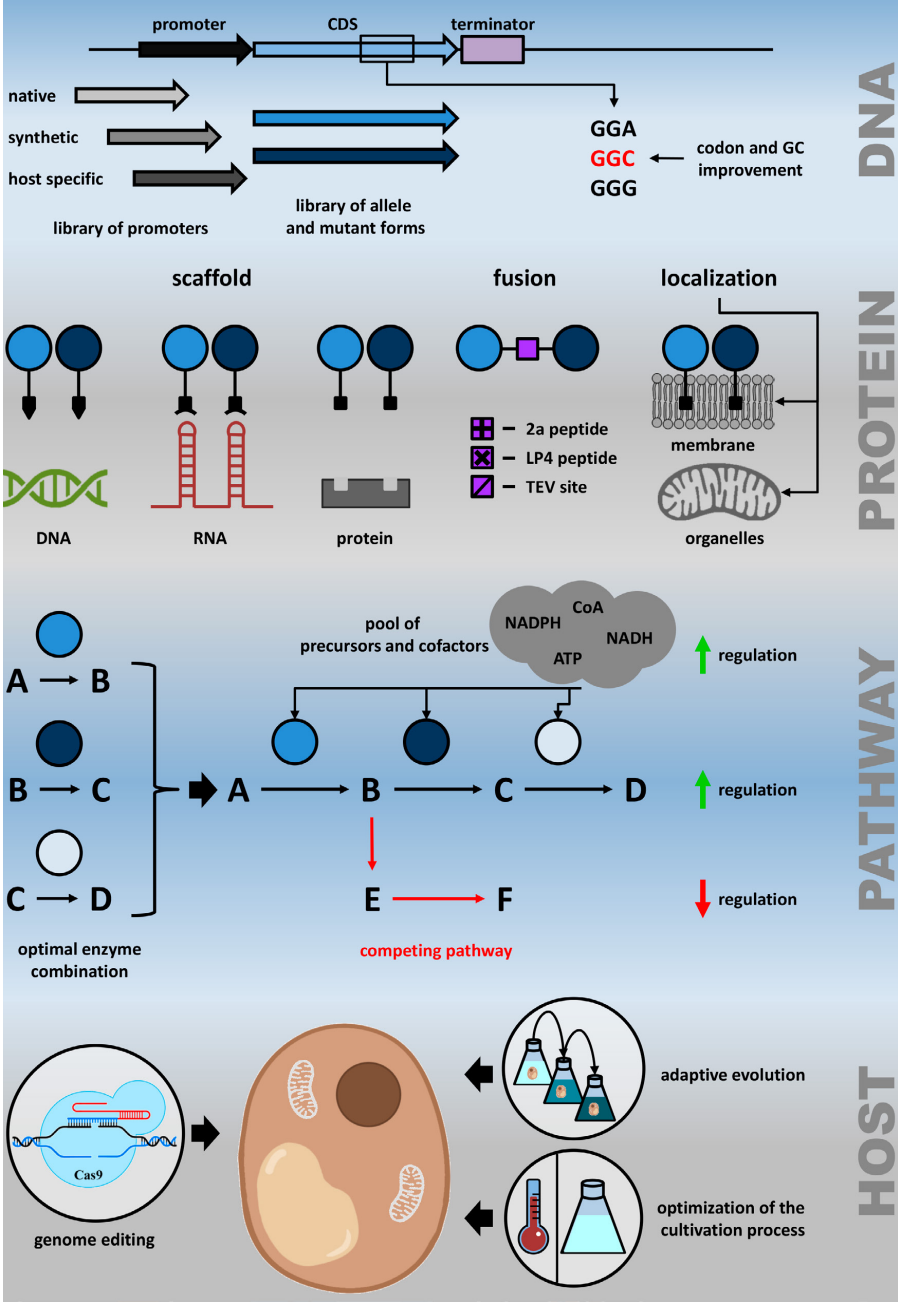
Schematic representation of heterologous pathway expression optimization strategies at the DNA, protein, pathway, and whole organism levels. DNA manipulation strategies include the selection of the best combinations of regulatory sequences, allele or mutant forms of coding sequences, and optimization of the GC- and codon content. The protein-level improvements are based on the engineering of scaffolds to ensure spatial proximity of the pathway enzymes or on engineering either direct or linker-separa‑ted fuses. Pathway-level optimization implies selection of the best enzyme combinations, upregulation of the desired pathways and downregulation of the competing native ones, and increasing the pool of substrates and cofactors. Finally, host-level improvement includes genome editing, adaptive evolution, and optimization of the cultivation process

Heterologous expression of natural product biosynthetic pathways is a multistage process each stage of which is fraught with difficulties. Problem accumulation has a strong impact on heterologous gene expression levels, resulting in low amounts or no production of target metabolites. Identification and elimination of metabolic bottlenecks are crucial for a successful expression of the heterologous pathway, thus significantly increasing the operation of the entire pathway. Bottleneck elimination depends on the physiological features of the host organisms, as well as on the properties of the metabolic pathway [55]. In this section, we will highlight the most frequent problems related to heterologous pathway expression and the main strategies for their resolution (*Fig. 3*).

**Product inhibition and metabolite toxic burden**

One of the common problems of heterologous expression is metabolic self-inhibition; i.e., the depression of enzyme activity by its own product. In the case of metabolic pathways, enzyme activity may be depressed at several stages, resulting in a measurable decrease in the biosynthesis rate and product yield depletion. The general solution to this problem is to substitute the feedback-regulated enzymes with their inhibition-resistant allele or mutant forms [56].

Another metabolite-related problem is the toxicity of the heterologous metabolic pathway products to the host cells [52]. In order to reduce the metabolic burden of the pathway expression on the cells of the host organism and to improve the yields of the desired metabolites, several measures might be taken depending on whether the toxicity is caused by intermediates or the final product. The negative impact of the intermediate may be decreased either by accelerating its conversion to the next compound [56] or by adaptive evolution of the host strain [57]. The adaptive evolution, a progressive increase in host resistance to the toxic metabolite under conditions of its constant burden, may also be applied in the case of final product toxicity [58]. Unfortunately, however, in some cases there is no obvious recipe for reducing the metabolic toxicity, and the only solution is to sacrifice the product yield to reduce the burden [59].

**Optimization of regulatory sequences**

Insufficient heterologous pathway expression may also be caused by the use of non-optimal regulatory sequences. There are two common approaches to promoter selection: whenever possible, the native promoters of the pathway genes are used or they are replaced with the host-specific regulatory sequence. The first approach is generally used when the host and the heterologous pathway source are phylogenetically close (e.g., two species belonging to the same genus) and the pathway is active in the source organism [60]. The second approach is more widespread, as it allows for the expression of evolutionarily distant metabolic pathways in common model hosts. The obvious drawback of this approach is that more complex molecular cloning procedures need to be applied, but today a set of various tools facilitating this step is available [34, 36, 61, 62].

Regulatory sequence fine-tuning might also be helpful in obtaining the optimal ratio of metabolic pathway enzymes [55]. For some pathways, the most efficient enzyme ratio is equimolar, which can be obtained, for instance, by using self-splitting fusion proteins linked to 2a peptides [63], LP4 peptides [64], dual-inteins [65], or the Tobacco Etch Virus recognition sequence [66, 67]. For the non-equimolar ratios, the use of promoters of varying strengths is a useful tool to fine-tune enzyme expression levels. At present, there exists a welldeveloped methodology for determining the required strength of promoters for each gene in the pathway [68, 60, 70]. The combination of the desired regulatory sequences with the coding ones can be easily achieved with the help of the Gibson assembly and Golden Gate Modular Cloning technologies [47, 71-74].

**GC content and codon-usage problems**

As mentioned above, a certain coding sequence can be obtained either directly from the source genome or synthesized chemically. The technologies of precise large-scale DNA synthesis, such as [75, 76], have recently become readily available and affordable. The additional benefit of chemical synthesis is that it allows one to modify the codon content in the coding sequences of the heterologous pathway according to the host preferences [2]. The improvement to the codon content is demonstrated to increase the expression of heterologous genes [77, 78, 79] and can be performed either manually with the help of databases [80] or through codon-optimization bioinformatic tools, which are freely available [75, 81]. Consequentially, codon optimization improves the GC content according to the host preferences, which allows for easier replication of heterologous DNA in the host organism and thus reduces the heterologous pathway burden.

**Optimization of the pathway enzymes combination**

The efficiency of a heterologous pathway does not linearly depend on the amount of gene copies. Initially, biosynthetic pathway metabolite production rises with increasing gene dosage; however, overexpression of heterologous proteins leads to a significant drop in the metabolic pathway output, since intracellular accumulation of metabolites can trigger cellular stress responses, and the metabolic efflux to the heterologous pathway cannot be balanced by the host cells [25]. Thus, addition of extra gene copies is useful only in case of genes encoding the rate-limiting enzymes, while changes in the copy number of other pathway genes have little or no effect on the final product titers [82]. The gene dosage approach has proved to be effective in heterologous β-carotenoid biosynthesis in *Yarrowia lipolitica *[83].

The most efficient heterologous pathway may comprise enzymes derived from diverse sources, with genes originating from several metabolic pathways or even different organisms [77]. In some cases, a combination of enzymes belonging to related biosynthetic pathways from different sources [42, 84] might be useful and sufficient, while in others an addition of auxiliary genes encoding activator proteins such as phosphopantetheine transferase is needed [85].

Spatial proximity of the enzymes’ active sites may increase the total rate of heterologous metabolite conversion and reduce the intermediate efflux and can be achieved by direct protein fusion or scaffolding. The advantage of scaffolds over direct fuses lies in preserving the enzyme amino acid sequences intact, which is generally better for the function of the protein. Three major scaffold types include the DNA scaffold, which is based on plasmids and allows one to easily change the distance between interacting proteins, the RNA scaffold, whose advantage is its small size, or the protein scaffold, a wide range of which is available [55].

Subcellular compartmentalization of heterologous pathway enzymes imposes spatial restriction on metabolite production. Fortunately, this issue can be resolved by co-localizing all the enzymes in the same compartment using well-characterized localization tags for mitochondria, endoplasmic reticulum, vacuole, nucleus, membrane, and peroxisome.

Membrane-associated enzymes impose the most stringent requirements on intracellular localization, thus often necessitating co-anchoring of all other metabolic pathway enzymes in the same membrane [86]. Several commercially available toolkits were designed to facilitate the correct colocalization of pathway enzymes in specific compartments in yeast [86] and plant chloroplasts [42].

As the ultimate aim of heterologous expression of metabolic pathways is the production of valuable secondary metabolites through a chain of enzymatic reactions, the sizes of individual heterologous proteins are irrelevant to the yield of the target product. The size of the expressed protein is characterized by the length of the coding sequence of the heterologous genes, as well as by the spatial restrictions imposed by cellular and subcellular compartmentalization of the heterologous pathways in the host cells. Thus, maximization of heterologous metabolite production is a multidimensional optimization problem to which the contribution of the pathway proteins efficiency prevails over their respective amounts and sizes.

**Metabolic flux and host pathway adjustment**

The substrate accessibility may dramatically influence the activity of the whole pathway. A preliminary metabolic flux analysis (MFA) based on NMR, mass spectroscopy, or other metabolomics approaches can facilitate planning of the heterologous pathway augmentation [10]. All the MFA methods are generally divided into two large groups: on-line methods that aim to quantify reactions rates (i.e., metabolic fluxes)* in situ*, and off-line methods based on sample collection [25]. Application of the MFA methods allows one to recognize the limiting steps of heterologous pathway expression, identity the branch-point metabolites and consequently optimize and redirect the metabolic flux towards the desired product. The main strategy of metabolite redistribution toward the heterologous pathway consists in tuning the expression levels of participating genes encoding both native and heterologous pathways [55].

This strategy is implemented by identification of the branch-point metabolite, common in both the host and heterologous pathways, and simultaneously upregulating the target compound pathway, and downregulating the rival native enzymes, while maintaining the balance between the two in order to preserve the host viability. The upregulation usually comprises activating [87] or doubling the host pathways [88], while downregulation is achieved by enzyme inhibition, transcript knock-down or complete removal of the genes of competing pathways [62].

This approach helps one to attain several objectives at once: enhance the final metabolite biosynthesis, increase the desired metabolic flux, and reduce the competing effluxes. For example, this method has yielded manifold improvements to the heterologous biosynthesis of alpha-santalene [89] and n-butanol [90] in yeast by adjusting the expression levels of the acetyl-CoA metabolism enzymes.

**Precursor accessibility**

Another key requirement for a sustainable and effective heterologous pathway expression is precursor availability. The deficit of ATP [91], CoA derivatives [92], NADH [93], NADPH [82], FMN [94], which are involved in the vast majority of biosynthetic pathways, was shown to be the limiting factor for heterologous metabolite production. In order to remove this bottleneck, the precursors and cofactors may be added exogenously or biosynthesized by the host. The latter approach is more efficient for poorly soluble or unstable substrates and can be achieved by activating host pathways, downregulating precursor competing pathways, or incorporating additional heterologous pathways [95, 96].

It is important to note that metabolite efflux to heterologous pathways can overlap and amplify the deleterious influence of heterologous products and inhibit the host primary metabolism [38]. No single strategy for solving this problem exists today; however, subcellular compartmentalization might be beneficial both for insulating branch-point metabolites from competing pathways and for sequestering the toxic end-products.

**Genome editing for heterologous pathway optimization**

Modern state-of-the-art genome editing technologies allow for unprecedented large-scale intervention into the host genome previously unattainable with other approaches. The heterologous pathway expression is assisted with such genome editing tools as RNAi [97], zinc-finger nucleases [98], DNA editing at replication forks [99], and the eminent CRISPR-Cas9 technology [10, 100, 101]. Genome editing also enables host genome stabilization by reducing the well-known problem of inactivation or recombination excision of heterologous genes [102]. The supreme form of genome editing is *de novo *synthesis of the host genomes containing nontypical sequences. This field is poorly developed for multicellular hosts, but several attempts to synthesize the yeast genome have been successful [103, 104].

**Optimization of the cultivation process**

When implementation of biotechnological methods has proved unsuccessful, adjustment of host cultivation protocols may yield the desired functioning of heterologous pathways. Adaptation of cultivation methods is a laborious and time-consuming process but may significantly improve the heterologous pathway expression [105]. For instance, the fed-batch cultivation, a protocol implying stepwise addition of the substrate to the growth medium, may be useful in the case of precursor toxicity [56, 106].

The problems related to host organism cultivation may also be solved by adjusting the host primary metabolism. An inspiring example is the recent creation of the novel strain of *P. pastoris *utilizing CO_2_ as a carbon source, which switches a heterotrophic organism to autotrophy [107].

## CONCLUSION

The valuable properties of many natural secondary metabolites, combined with their low levels of production in native organisms, translate into an increasing relevance of the development of heterologous expression techniques. This review has analyzed and summarized the common limiting factors impeding heterologous expression in eukaryotic hosts and suggested several important avenues for improvement, which involve applying the most advanced molecular biology tools to each problem. Since heterologous metabolic pathway expression is not a single method but a plethora of various approaches, no universal advice to researchers, who are taking their first steps in this area, exists. Nevertheless, the numerous encouraging examples of heterologous pathway expression create a high degree of confidence as to the future of the field. Thus, as demand for the heterologous expression of complex metabolic pathways rises, the principal tools and techniques of metabolic engineering examined here may guide researches in their quest to create successful and productive heterologous expression systems and advance the application of eukaryotic hosts.
